# Effectiveness of Otolith Repositioning Maneuvers and Vestibular Rehabilitation exercises in elderly people with Benign Paroxysmal Positional Vertigo: a systematic review^☆^

**DOI:** 10.1016/j.bjorl.2017.06.003

**Published:** 2017-06-29

**Authors:** Karyna Figueiredo Ribeiro, Bruna Steffeni Oliveira, Raysa V. Freitas, Lidiane M. Ferreira, Nandini Deshpande, Ricardo O. Guerra

**Affiliations:** a Universidade Federal do Rio Grande do Norte (UFRN), Programa de Pós-Graduação em Ciências da Saúde, Natal, RN, Brazil; b Universidade Federal do Rio Grande do Norte (UFRN), Departamento de Fisioterapia, Natal, RN, Brazil; c Universidade Federal do Rio Grande do Norte (UFRN), Programa de Pós-Graduação em Saúde Pública, Natal, RN, Brazil; d Queen's University, Faculty of Health Sciences, School of Rehabilitation Therapy, Kingston, Canada; e Universidade Federal do Rio Grande do Norte (UFRN), Programa de Pós-Graduação em Fisioterapia, Natal, RN, Brazil

**Keywords:** Benign Paroxysmal Positional Vertigo, Elderly, Vertigo, Dizziness, Rehabilitation, Vertigem posicional paroxística benigna, Idosos, Vertigem, Tontura, Reabilitação

## Abstract

**Introduction:**

Benign Paroxysmal Positional Vertigo is highly prevalent in elderly people. This condition is related to vertigo, hearing loss, tinnitus, poor balance, gait disturbance, and an increase in risk of falls, leading to postural changes and quality of life decreasing.

**Objective:**

To evaluate the outcomes obtained by clinical trials on the effectiveness of Otolith Repositioning Maneuver and Vestibular Rehabilitation exercises in the treatment of Benign Paroxysmal Positional Vertigo in elderly.

**Methods:**

The literature research was performed using PubMed, Scopus, Web of Science and PEDro databases, and included randomized controlled clinical trials in English, Spanish and Portuguese, published during January 2000 to August 2016. The methodological quality of the studies was assessed by PEDro score and the outcomes analysis was done by critical revision of content.

**Results:**

Six studies were fully reviewed. The average age of participants ranged between 67.2 and 74.5 years. The articles were classified from 2 to 7/10 through the PEDro score. The main outcome measures analyzed were vertigo, positional nystagmus and postural balance. Additionally, the number of maneuvers necessary for remission of the symptoms, the quality of life, and the functionality were also assessed. The majority of the clinical trials used Otolith Repositioning Maneuver (*n* = 5) and 3 articles performed Vestibular Rehabilitation exercises in addition to Otolith Repositioning Maneuver or pharmacotherapy. One study showed that the addition of movement restrictions after maneuver did not influence the outcomes.

**Conclusion:**

There was a trend of improvement in Benign Paroxysmal Positional Vertigo symptomatology in elderly patients who underwent Otolith Repositioning Maneuver. There is sparse evidence from methodologically robust clinical trials that examined the effects of Otolith Repositioning Maneuver and Vestibular Rehabilitation exercises for treating Benign Paroxysmal Positional Vertigo in the elderly. Randomized controlled clinical trials with comprehensive assessment of symptoms, quality of life, function and long-term follow up are warranted.

## Resumo

**Introdução:**

A vertigem posicional paroxística benigna é altamente prevalente em idosos. Esta condição está relacionada a vertigem, perda auditiva, zumbido, equilíbrio precário, distúrbios da marcha e aumento do risco de quedas, levando a mudanças posturais e redução da qualidade de vida.

**Objetivo:**

Avaliar os desfechos obtidos por ensaios clínicos sobre a eficácia da Manobra de Reposicionamento de Otólitos e de exercícios de Reabilitação Vestibular no tratamento de vertigem posicional paroxística benigna em idosos.

**Método:**

A pesquisa da literatura foi realizada usando bancos de dados do PubMed, Scopus, Web of Science e PEDro e incluiu ensaios clínicos controlados randomizados em inglês, espanhol e português, publicados de janeiro de 2000 a agosto de 2016. A qualidade metodológica dos estudos foi avaliada pelo escore PEDro e a análise dos desfechos foi realizada por revisão crítica do conteúdo.

**Resultados:**

Seis estudos foram totalmente revisados. A idade média dos participantes variou entre 67,2-74,5 anos. Os artigos foram classificados de 2 a 7/10 pelo escore PEDro. As principais medidas de desfecho analisadas foram vertigem, nistagmo posicional e equilíbrio postural. Além disso, o número de manobras necessárias para a remissão dos sintomas, a qualidade de vida e a funcionalidade também foram avaliados. A maioria dos ensaios clínicos usou Manobra de Reposicionamento de Otólitos (n = 5) e 3 artigos realizaram exercícios de RV, além de Manobra de Reposicionamento de Otólitos ou farmacoterapia. Um estudo mostrou que a adição de restrições de movimento após a manobra não influenciou os resultados.

**Conclusão:**

Houve uma tendência de melhora na sintomatologia da vertigem posicional paroxística benigna em pacientes idosos submetidos à Manobra de Reposicionamento de Otólitos. Existem evidências escassas de ensaios clínicos metodologicamente robustos que examinaram os efeitos dos exercícios de Manobra de Reposicionamento de Otólitos e Reabilitação Vestibular para o tratamento da vertigem posicional paroxística benigna nos idosos. Ensaios clínicos controlados randomizados com avaliação abrangente de sintomas, qualidade de vida, função e acompanhamento de longo prazo são justificados.

## Introduction

Dizziness is one of the most common symptoms in elderly people and it is considered a geriatric syndrome.^1^ Among the causes of dizziness, Benign Paroxysmal Positional Vertigo (BPPV) is the most frequent vestibular disorder, affecting approximately 20% of patients presenting this symptom. BPPV is highly prevalent in elderly, probably due to senile degenerative changes.^2^, ^3^ Diagnosis of BPPV is confirmed using Dix-Hallpike test, and it is classified as objective when nystagmus is observed during the test, or subjective when there is vertigo without nystagmus.^4^ Female patients have been shown to be most affected by BPPV, which may be justified by the fact that the homeostasis of labyrinthine fluids may be compromised by female hormones decreasing from climacteric phase.^5^

Prevalence of BPPV is estimated at 25% in elderly people over 70 years with complaints about dizziness and this symptom persists for more than one year.^6^, ^7^, ^8^ Vertigo is reported as the main complain of BPPV patients and may be associated to hearing loss, tinnitus, poor balance, gait disturbance, and an increase in risk of falls.^9^ Patients with BPPV restrict their activities in order to avoid crises due vertiginous symptomatology, leading to postural changes and quality of life decreasing.^8^, ^10^ Such movement restrictions associated to comorbidities and high prevalence of BPPV in elderly usually result in functional loss and inability.^11^, ^12^

BPPV also increases incidence of falls in older patients, as well as chance of fracture, head traumas (concussion), hospitalizations and depression.^13^ Elderly with BPPV show worse scores in functional tests due to coexistence of multiple morbidities, fear of falling that characterizes geriatric population and the senescence of vestibular system usually found in this population, which may further damage postural balance in these individuals.^13^, ^14^ Furthermore, static and dynamic postural control in elderly patients with vestibulopathies is damaged, which may contribute to a functional limitation and greater low balance confidence regarding falls in this population.^3^, ^15^, ^16^, ^17^, ^18^

Vertigo and other associated symptoms are triggered by the displacement of statocone (otoconia) fragments from the utricle macula. The statocone freely float in the endolymph of one or more semicircular canals which become sensitive to changes in head position.^19^ For these reasons, BPPV is mainly treated by Otolith Repositioning Maneuvers (ORM) in order to move the otoconia out of the canal and lead it back to the vestibule. However, some authors indicate that ORM is not sufficient to improve or recover postural stability in elderly people with BPPV.^20^, ^21^, ^22^ Other non-pharmacological intervention for patients with balance disturbances is the Vestibular Rehabilitation (VR) exercises, which includes vestibular adaptation, habituation and substitution exercises, and patient education.^23^, ^24^, ^25^

Although the use of ORM and VR exercises on treating BPPV are commonly proposed in the literature, it was observed that the majority of studies include a huge age variation in their experimental designs and intervention forms. Therefore, the present review aimed to evaluate the outcomes from randomized controlled clinical trials about the effectiveness of ORM and VR exercises in the treatment of BPPV in elderly people.

## Methodology

For the present systematic review, the scientific question: “What is the effectiveness of ORM and/or VR exercises in the treatment of BPPV in elderly people?”, was established using the PICO strategy.^26^ The P-Patient component of the PICO strategy refers to elderly with BPPV; the I-Intervention refers to ORM and/or VR exercises; and the O-Outcomes is related to vertigo, dizziness, and postural balance. The component C-Comparison was excluded from the study because there is no comparison between interventions. Bibliographic research was performed during September 2016 concomitantly in PubMed, Scopus, Web of Science and PEDro databases. It was limited to Portuguese, English and Spanish language papers which were published from January, 2000 to August, 2016. The strategy used was the combination of the descriptors “benign paroxysmal positional vertigo” AND “physical therapy modalities” OR “rehabilitation” OR “exercise therapy” AND “vertigo” OR “dizziness” OR “postural balance”. After this process, only two key-words were combined (“Benign paroxysmal positional vertigo” AND “therapy”; “Benign Paroxysmal Vertigo” AND “Exercises”; “Benign Paroxysmal Positional Vertigo” AND “Treatment”; “Benign Paroxysmal Positional Vertigo” AND “Physical Therapy”). Duplicate articles among the databases were excluded.

The following inclusion criteria were applied: (1) Participants with an average age of 65 years and over; (2) Individuals with BPPV and; (3) Interventions by VR exercises and/or ORM. The studies were excluded if they were non-randomized clinical trials, qualitative studies, and studies with pharmacological or surgical interventions without association to VR exercises and/or ORM.

The construction of this systematic review was guided by the criteria of the Preferred Reporting Items for Systematic Reviews and Meta-Analysis (PRISMA statement).^27^ The methodological quality of selected studies was assessed by PEDro score, which is comprised of 11 criteria about the internal validity and interpretation of clinical trials.^28^ The score attributes 1 point for each criterion presented by the study. However, the first criterion (eligibility criterion) is not counted. Therefore, the closer the score is to 10 obtained by the study, the better is its methodological quality and data reproducibility. Each article score is given by trained specialists and it is available in PEDro database.^29^

The studies selected for a full review were analyzed by two independent researchers and the disagreements between them were solved in consensus with assistance of a third evaluator who analyzed the divergent questions.

## Results

**Figure 1:**
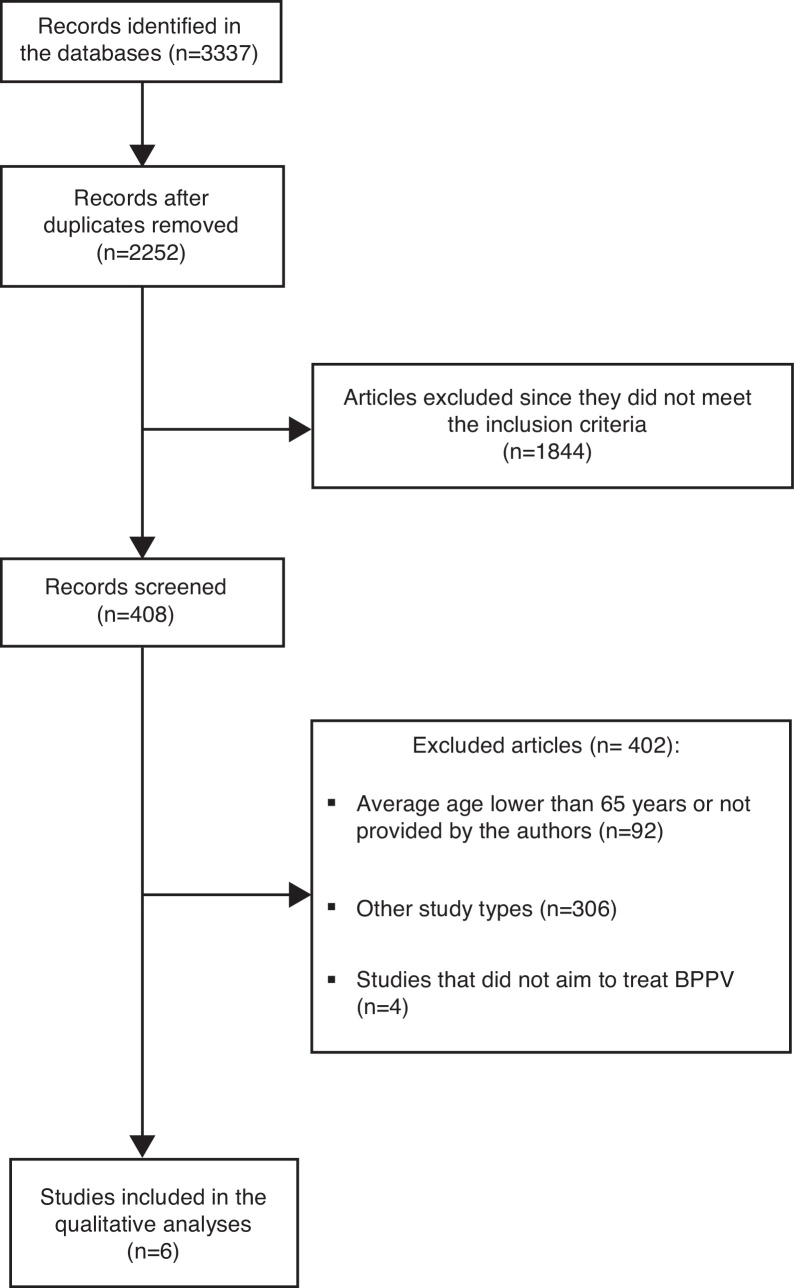
Fluxogram for the selection of articles.

The research performed by the health descriptors and keywords resulted in 3337 articles, but 1085 studies were duplicates. Out of these, 1844 studies were excluded since they did not meet the inclusion criteria. Four hundred and eight abstracts were scrutinized. By reading the abstracts, it was found that 306 were not randomized controlled clinical trials, 92 had average ages lower than 65 years or did not show the average age in full text and 4 did not include BPPV as a sample characteristic (Fig. 1). Thereby, 6 randomized controlled clinical trials passed the criteria required for this review and were selected for critical analysis of their content. The synopsis of main data from the reviewed articles is displayed in Table 1. Table 2 shows that PEDro score ranged from 2 to 7.

**Table 1:** Synopsis of data from randomized controlled clinical trials about the effectiveness of Otolith Repositioning Maneuvers (ORM) and Vestibular Rehabilitation (VR) exercises in the treatment of Benign Paroxysmal Positional Vertigo (BPPV) in elderly people.

Author Year Country	Sample	Age (mean-median) in years	Outcome measures	Intervention	Results
Angeli et al. (2003) United States^(PEDro:^^4/10)^	EG: 28 CG: 19 PC-BPPV	EG: 74.5 ± 4.5 CG: 74.2 ± 3.4	(1) Vertigo: - Reported during Dix-Hallpike test (2) Positional nystagmus: - Electronystagmo-graphy during Dix-Hallpike test.	**1st Phase:** **-** EG: ORM (Epley maneuver); CG: no intervention. *Number of maneuvers*: 1–3. *Follow-up*: 1 month. **2nd Phase:** **-** CG: Participants in CG who did not achieve spontaneous remission of symptoms received ORM (after 1 month). - The participants from both groups who did not get remission of symptoms, underwent a supervised VR exercises program. *VR frequency*: 2 or 3 times a week. *Follow-up*: 4–6 weeks.	**1st Phase:** - 64% of patients from EG obtained negative Dix-Hallpike test (no vertigo or nystagmus) compared to only 5% in CG (*p* < 0.001). There was no difference in cure rate between the CG subgroup and the experimental group after maneuver performance (*p* = 0.553). **2nd Phase:** **-** 18 patients received personalized VR. Seven patients had total remission of symptoms and/or nystagmus (negative Dix-Hallpike), 6 continued to present a positive test and 5 did not conclude the study. - At the end, 77% of patients obtained success in treatment.
Resende et al. (2003) Brazil^(PEDro:^^4⧸10)^	EG: 8 CG: 8 Did not specify the canal	EG: 70.5 (61–82) CG: 69.3 (60–78)	(1) Functionality (VDADL)	EG: Cawthorne and Cooksey exercises protocol and pharmacotherapy (Gingko-Biloba–40 mg de 12/12 h); *VR frequency: 2 sessions a week, during 5 weeks.* CG: Pharmacotherapy. *Follow-up: 5 weeks (EG) and 30 days (CG).*	Significant decrease in VDADL scores in EG (*p* < 0.01). There were no differences in the final score of VDADL in CG compared to pre-treatment phase. Significant benefit to EG compared to CG (*p* < 0.009).
Salvinelli et al. (2004) United Kingdom^(PEDro:^^4⧸10)^	G1: 52 G2: 52 G3: 52 PC-BPPV	G1: 73 (70–78) G2: 74.5 (71–80) G3: 75 (72–79)	(1) Vertigo: - Reported during Dix-Hallpike test (2) Functionality (VDADL)	- G1: ORM (SLM). *Number of maneuvers*: 1–3 consecutive maneuvers per week until symptom resolution. - G2: Calcium antagonists (10 mg/d of Flunarizine before sleeping for 60 days); - G3: no treatment. *Follow-up*: 6 months after the end of each treatment.	- G1: 94.2% of vertigo remission after 3 maneuvers and 3.8% of recurrence in 6 months. - G2: 57.7% of symptoms remission; 5.8% of recurrence in 6 months; - G3: 34.6% had spontaneous remission of symptoms; 21.1% of recurrence in 6 months; - A statistically significant post-treatment improvement in activities of daily living and in quality of life was noticed (*p* < 0.001).
André et al. (2010) Brazil^(PEDro:^^2⧸10)^	G1: 23 G2: 15 G3: 15 PC-BPPV Ductolithiasis	67.2 (60–91) Authors did not provide the median age by group	(1) Dix-Hallpike test; (2) Clinical aspects and symptoms: referred by Brazilian DHI questionnaire.	- G1: ORM (Epley maneuver) + neck brace + Postural restrictions for 48 hours after maneuver; - G2: ORM (Epley maneuver); - G3: ORM (Epley maneuver) + Mini vibrator *Number of maneuvers*: one per session until complete remission of vertigo. *Follow up*: time between evaluations was not informed.	- Number of maneuvers ranged from 1 to 3 in all groups. No difference was found between groups; - Statistically significant difference was observed in all aspects evaluated by DHI after treatment in all groups; - Significant improvement on physical aspects of G1 after treatment when compared to G2 (*p* = 0.009); - Independent of the procedure after maneuver the ORM was effective based on DHI score.
Ribeiro et al. (2016) Brazil ^(PEDro:^^7⧸10)^	EG: 7 CG: 7 PC-BPPV	EG: 69 (65–78) CG: 73 (65–76)	(1) Dix-Hallpike test; (2) Vertigo–evaluated by VAS; (3) Number of maneuvers.	EG: ORM (Epley maneuver) + VR CG: ORM (Epley maneuver) *Number of maneuvers*: 1 to 3 per session. *VR frequency*: 2 times a week. *Follow-up:* 13 weeks	- The median for the number of comorbidities in the EG was 4 (3–6) and 4 (2–6) in the CG, while the median number of drugs used was 3 (1–6) in the EG and 3 (3–7) in the CG. - The Dix-Hallpike test was negative for all seniors in the EG after 13 weeks, while the CG showed treatment failure in 28.6% patients. - There was significant improvement gradient, with a progressive decrease in the number of maneuvers necessary for the treatment in EG (*p* < 0.001). - The recurrence rate was higher, yet not statistically significant, in the CG in the 13 week period.
Ribeiro et al. (2016) Brazil^(PEDro:^^7⧸10)^	EG: 7 CG: 7 Did not specify the canal	EG: 69 (65–78) CG: 73.5 (72–76)	(1) Postural balance–evaluated by computadorized posturography and DGI; (2) Vertigo–evaluated by VAS; (3) Quality of life–evaluated by DHI.	EG: ORM (Epley maneuver) + balance VR CG: ORM (Epley maneuver) *Number of maneuvers*: 1–3 per session. *VR frequency*: 2 times a week. *Follow-up*: 13 weeks	- No differences between groups were found regarding all standing balance aspects. However, there was a within group improvement in standing balance in all tests in experimental group. - Concerning dynamic balance, there were significant differences between groups in the majority of the tests. In within group comparisons, the experimental group's all dynamic balance parameters significantly improved. Conversely, no significant differences were found regarding dynamic balance in the control group. - There were no significant differences in dizziness symptoms and quality of life between groups. However, both groups showed intragroup significant improvement for both outcome measures.

EG, experimental group; CG, control group; G1, Group 01; G2, Group 02; G3, Group 03; DHI, Dizziness Handicap Inventory; DGI, Dynamic Gait Index; VAS, Visual Analog Scale; VDADL, Vestibular Disorders Activities of Daily Living scale; PC-BPPV, Benign Paroxysmal Positional Vertigo of the posterior canal; VR, Vestibular Rehabilitation; SLM, Semont Liberatory Maneuver.

**Table 2:** Methodological analysis by PEDro score of clinical trials about the effectiveness of Otolith Repositioning Maneuvers (ORM) and Vestibular Rehabilitation (VR) exercises in the treatement of Benign Paroxysmal Positional Vertigo (BPPV) in elderly people.

	Angeli et al. 2003	Resende et al. 2003	Salvinelli et al. 2004	André et al. 2010	Ribeiro et al. 2016	Ribeiro et al. 2016
1. Eligibility criteria were specified	Yes	Yes	Yes	Yes	Yes	Yes
2. Subjects were randomly allocated to groups	Yes	Yes	No	Yes	Yes	Yes
3. Allocation was concealed	No	No	No	No	Yes	Yes
4. The groups were similar at baseline regarding the most important prognostic indicators	Yes	Yes	No	No	Yes	Yes
5. There was blinding of all subjects	No	No	No	No	No	No
6. There was blinding of all therapists who administered the therapy	No	No	No	No	No	No
7. There was blinding of all assessors who measured at least one key outcome	No	No	No	No	Yes	Yes
8. Measures of at least one key outcome were obtained from more than 85% of the subjects initially allocated to groups	Yes	No	Yes	No	Yes	Yes
9. All subjects for whom outcome measures were available received the treatment or control condition as allocated or, where this was not the case, data for at least one key outcome was analyzed by “intention to treat”	No	No	No	No	No	No
10. The results of between-group statistical comparisons are reported for at least one key outcome	Yes	Yes	Yes	Yes	Yes	Yes
11. The study provides both point measures and measures of variability for at least one key outcome	No	Yes	Yes	No	Yes	Yes
Score	4/10	4/10	3/10	2/10	7/10	7/10

Among the six selected studies, the sample number varied from 14 to 156 patients, totaling 300 participants. The average age varied from 67.2 to 74.5 years. One article^30^ used Semont Liberatory Maneuver (SLM) as the intervention, while four studies used Epley maneuver^20^, ^31^, ^32^, ^33^ and one study implemented movement restrictions after the ORM and used a cervical collar and a mini-vibrator applied on the mastoid of the affected side.^31^ Four studies applied VR exercises^20^, ^32^, ^33^, ^34^ and two articles applied pharmacotherapy.^30^, ^34^ Table 1 provides details about the intervention strategies used in each study.

The analyzed variables in the selected studies included: vertigo,^20^, ^30^, ^32^, ^33^ positional nystagmus^20^ and postural balance,^33^ the number of necessary maneuvers for remission of symptoms^31^, ^32^ and the quality of life by Dizziness Handicap Inventory (DHI) score,^31^, ^33^ and functionality by the Vestibular Disorders Activities of Daily Living scale (VDADL).^30^, ^34^

## Discussion

The increment of older population around the world will require special attention government health services. Comorbidities related to the aging process underlined by the deficit in physiological, cognitive and social functions, contribute to the development of diseases in multiple biological systems. BPPV is the most common cause of vestibular vertigo and one of the otoneurological conditions that has the highest prevalence in the geriatric population, leading to strong impact on the health and quality of life of these individuals.^35^ Non-pharmacological alternatives for its treatment, including the ORM, represent an important therapeutic opportunity as a result of absence of side effects risks commonly seen in older people.

Among the studies evaluated by PEDro score, the highest classification was 7/10. Nevertheless, the two articles evaluated with this score had a low sample (only 7 in each group). These findings warrant the need to conduct randomized controlled and blinded clinical trials in elderly people with BPPV, with robust methodology. In the majority of studies, patients presented BPPV of posterior canal,^20^, ^30^, ^31^ which according to literature is the most prevalent diagnosis.^18^, ^22^, ^36^ Two studies did not specify the affected canal.^33^, ^34^

As for the ORM intervention in elderly, most studies applied the modified Epley maneuver.^20^, ^31^, ^32^, ^33^ No study used the classic Epley maneuver, but the study of André et al.^31^ added a mini-vibrator on the mastoid process of the affected side. All the studies that used modified Epley maneuver described improvements in BPPV symptomatology, mainly for vertigo, dizziness and nystagmus. These findings agree with current literature, which places the Epley maneuver first in the treatment of BPPV of posterior canal. Fife et al.^37^ classified the Epley maneuver for otolith repositioning as “Recommendation level A.” In other words, the therapy is effective and safe and must be offered to patients with BPPV of posterior canal of all ages.

The literature indicates SLM as the treatment for cupulolithiasis of anterior and posterior canals.^29^, ^36^ One article mentioned having performed it. However, this study performed SLM without clarifying if patients had cupulolithiasis or canalithiasis,^30^ with the exception being André et al.^31^ that clarified their sample as BPPV of posterior canal with ductolithiasis (canalithiasis). In the study conducted by Salvinelli et al.,^30^ the group that underwent SLM demonstrated a significantly superior percentage of symptom remission compared to the one that used only pharmacological intervention with Flunarizine^®^ (10 mg/d before sleeping during 60 days). Furthermore, symptom recurrence rate was also lower in the maneuver group after 6 months.

The effect of movement restrictions after ORM has been a target intervention of one research, which assessed the efficacy of this practice after implementing the maneuver.^31^ André et al.^31^ used a neck brace in one group, in addition to movement restriction instructions. According to the findings of this study, the movement restrictions after ORM do not influence outcomes. These data are in concordance with the international guideline elaborated by Fife et al.,^37^ which classified movement restrictions as “Recommendation U,” therefore, there are not enough data to support its use in clinical practice.

Only the studies of André et al.^31^ and Ribeiro et al.^33^ evaluated quality of life after management of BPPV. They concluded that the Epley maneuver improves patient's DHI score, which indicates that this procedure is effective to decrease the impact of the vertiginous symptoms. Similar findings are found after the treatment of people with VPPB in other age groups.^38^

According to number of maneuvers, the studies that used ORM varied from 1 to 3 maneuvers in general. Four studies applied 1–3 maneuvers in the same session.^20^, ^32^, ^33^ Two studies performed one maneuver per session with a range of 1–3 maneuvers (sessions) among groups and concluded that the ORM was effective for symptom remission.^30^, ^31^ Korn et al.^39^ suggest that consecutive maneuvers in the same session seem to be more effective than only one maneuver per session. On the other hand, Kasse et al.^12^ conducted a quasi-experimental study in 33 older patients with BPPV and performed the ORM only once per session, then repeated weekly until symptoms and nystagmus disappeared (remission), and also concluded that ORM was effective. Therefore, despite ORM being an effective intervention for BPPV in elderly regardless of the protocol performed by the studies, it is not possible to propose a standard number of maneuvers, or if they should be performed in the same session or in different ones.

Only one study that did not use ORM as therapeutic proposal was found,^34^ but its main intervention was VR exercises for an elderly sample. They applied Cawthorne and Cooksey exercise protocols associated to Gingko-Biloba in experimental group and only drug intervention in the control group. The authors obtained significant improvement referring to functionality by performing therapeutic exercises; however, there are not reports denoting improvements in BPPV symptoms and signs (vertigo and nystagmus). Angeli et al.^20^ also used VR exercises in elderly people with BPPV and randomly assigned the patients into 2 groups in the first part of the study: ORM and no treatment. After one month, those patients who did not respond to treatment were enrolled in the second part of the study and were treated with an individualized combination of ORM and VR, and then reevaluated 3 months later. The authors concluded that the maneuvers are more effective compared to no treatment, and VR exercises can be added to ORM to improve results in the treatment of BPPV in elderly people.

Furthermore, Angeli et al.^20^ observed a considerable rate of symptoms recurrence in elderly who only underwent ORM and they suggest that VR exercises can decrease recurrence rate of BPPV. They stated that this protector effect can be more evident in elderly people.^20^ The study conducted by Ribeiro et al.^32^ also aimed to verify the recurrence rate between the group that performed only ORM and the group that performed VR associated to ORM, but there was not statistically significant difference between the groups. Some studies have shown that VR exercises in younger and older patients with BPPV are more effective alone when compared to no treatment or placebo treatment.^40^, ^41^ Silva et al.^24^ analyzed two international guidelines^37^, ^42^ and considered VR exercises as possibly effective, becoming a secondary option in the treatment of BPPV.

The majority of selected articles provide short-term results ranging from 4 weeks to 13 weeks post-follow up. Only the study of Salvinelli et al.^30^ presented a longer follow up of six months and they observed a higher rate of symptom recurrence (21.1%) in none treatment group compared to the ORM group (3.8%).^43^ Ganança et al.^18^ reevaluated an elderly sample in their quasi experimental study after one year of successful ORM and observed BPPV recurrence rate of 21.5%. According to Brandt et al.^44^ and Simhadri et al.,^45^ the recurrence rate in treated cases varies between 10% and 80%. This variability found in literature in relation to BPPV recurrence rate may occur due to methodology differences among studies. Ganança et al.^18^ believe that the longer the follow up is, the higher the proportion of recurrence rate of BPPV cases is. Although results of BPPV treatment are encouraging, the recurrence of dizziness, particularly in the elderly, is very high and new studies with long term follow up would be necessary for these patients.^6^, ^7^, ^8^

Although postural balance is often impaired in the elderly, especially in those with vestibular disorders,^7^, ^18^ it was observed that only one of the studies evaluated standing and/or dynamic postural balance in this population. Ribeiro et al.^33^ showed the effects of the VR in the postural balance in elderly people with BPPV. The exercises included oculomotor exercises (VOR × 1), habituation exercises (repeated head and trunk movements), standing and dynamic balance training, along with lower-limb muscles strengthening. For each exercise prescription, a universal set of 10 modifiers and progression patterns were followed to make the exercises more challenging.^46^ It was found that the group which performed the VR improved the dynamic balance when compared to the control group that performed only the ORM. Nonetheless, quasi-experimental studies demonstrate the efficacy of ORM in improving balance in the elderly population.^12^, ^21^, ^47^ Ganança et al.^18^ performed a study on elderly people and concluded that the number of falls decreased in consequence of vertigo and nystagmus remission after a maneuver. The review's limitations include a high standard deviation in average age, which means there were also younger persons in some studies. Furthermore, postural balance and functionality were barely evaluated in the studies despite of the clinical importance in the elderly population, mainly in those who suffer from dizziness.

All the studies that used ORM in elderly people with BPPV showed a trend of improvement in their symptomatology, mainly for vertigo, dizziness and nystagmus. Regardless of the procedures performed by the studies, there was a huge range in the number of maneuvers to obtain a negative Dix-Hallpike test, thus it is not possible to propose an ORM standard protocol. There is a lack of robust methodological studies that used VR in this population, thus it is not possible to conclude that this intervention is effective. It seems that the movement restrictions after maneuver do not influence results. Overall, there is sparse evidence from methodologically robust clinical trials that have examined the effects of ORM and VR exercises for treating BPPV in the elderly population. Randomized controlled clinical trials with comprehensive assessment of symptoms, quality of life, function and long-term follow up are warranted.

## Conflicts of interest

The authors declare no conflicts of interest.
